# 433. Thromboinflammation in COVID-19 and association with death in patients on ECMO

**DOI:** 10.1093/ofid/ofad500.503

**Published:** 2023-11-27

**Authors:** Andrew Platt, Viviane Callier, Zonghui Hu, Seth Warner, Syndey R Stein, Megan Anders, Emily Ricotta, Trevor M Stantliff, Jocelyn Wu, Nicole Hays, Katherine Raja, Ryan Curto, Madeleine Purcell, Sabrina Ramelli, Marcos J Ramos-Benitez, Izabella Lach, James Dickey, Jeffrey R Strich, Kevin M Vannella, Miranda Gibbons, Ali Tabatabai, Kapil K Saharia, Ronson Madathil, Joseph Rabin, Alison Grazioli, Dean Follmann, Daniel S Chertow

**Affiliations:** National Institute of Allergy and Infectious Diseases, Washington, DC; Frederick National Laboratory for Cancer Research, Bethesda, Maryland; National Institute of Allergy and Infectious Disease, Bethesda, Maryland; Clinical Center, NIH, Bethesda, Maryland; Clinical Center, NIH, Bethesda, Maryland; University of Maryland Medical Center, Baltimore, Maryland; National Institute of Allergy and Infectious Diseases, Washington, DC; Clinical Center, NIH, Bethesda, Maryland; University of Maryland School of Medicine, Baltimore, Maryland; University of Maryland School of Medicine, Baltimore, Maryland; University of Maryland School of Medicine, Baltimore, Maryland; University of Maryland School of Medicine, Baltimore, Maryland; University of Maryland School of Medicine, Baltimore, Maryland; National Cancer Institute, Bethesda, Maryland; Clinical Center, NIH, Bethesda, Maryland; Clinical Center, NIH, Bethesda, Maryland; Clinical Center, NIH, Bethesda, Maryland; Clinical Center, National Institutes of Health, Bethesda, Maryland; Clinical Center, NIH, Bethesda, Maryland; University of Maryland Medical Center, Baltimore, Maryland; University of Maryland St. Joseph Medical Center, Baltimore, Maryland; University of Maryland School of Medicine, Baltimore, Maryland; Wellspan Health York Hospital, Baltimore, Maryland; University of Maryland School of Medicine, Baltimore, Maryland; University of Maryland School of Medicine, Baltimore, Maryland; National Institute of Allergy and Infectious Disease, Bethesda, Maryland; Clinical Center, National Institutes of Health, Bethesda, Maryland

## Abstract

**Background:**

Critical illness in COVID-19 may require mechanical support with Extra-Corporeal Membrane Oxygenation (ECMO). Thromboinflammation, the interaction of thrombotic and inflammatory pathways, is seen in many conditions including sepsis and acute respiratory distress syndrome (ARDS) and may drive poor outcomes in critically ill patients. We sought to characterize thromboinflammation in critically ill patients with COVID-19 on ECMO and determine if it was associated with death.

**Methods:**

74 consecutive patients with COVID-19 who were cannulated and supported on ECMO at a single academic center between May 2020 and February 2021 were included in this study. Demographic, clinical and laboratory variables were collected daily and a panel of 16 thromboinflammatory biomarkers were measured in plasma on days 1, 3, 5, 8, 11, 15, 22, 29, and 40 of cannulation in a bead-based multiplex assay. Digital droplet PCR was used to measure plasma SARS-CoV-2 nucleocapsid RNA. Non-parametric mixed-effects models were used to estimate biomarker trajectories, and Cox regression was used to determine hazard ratios while controlling for demographics.

**Results:**

Male sex, smoking status, high bilirubin, and low ratios of the partial pressure of oxygen in arterial blood to the fraction of inspired oxygen (P/F ratio) were associated with an increased risk of death. Non-parametric mixed-effect regression indicated angiopoietin, IL-1a, IL-1b, IL-6, IL-8, Syndecan-1, VCAM1, and ICAM were higher in patients who died. In Cox regression models IL-6, IL-8, S100A8, thrombomodulin, and von Willebrand factor among others were associated with hazard of death or decannulation when controlling for sex, smoking status, and time from symptom onset to cannulation. Viral RNA was highest in patients immediately after cannulation but did not predict death.

**Figure 1:**
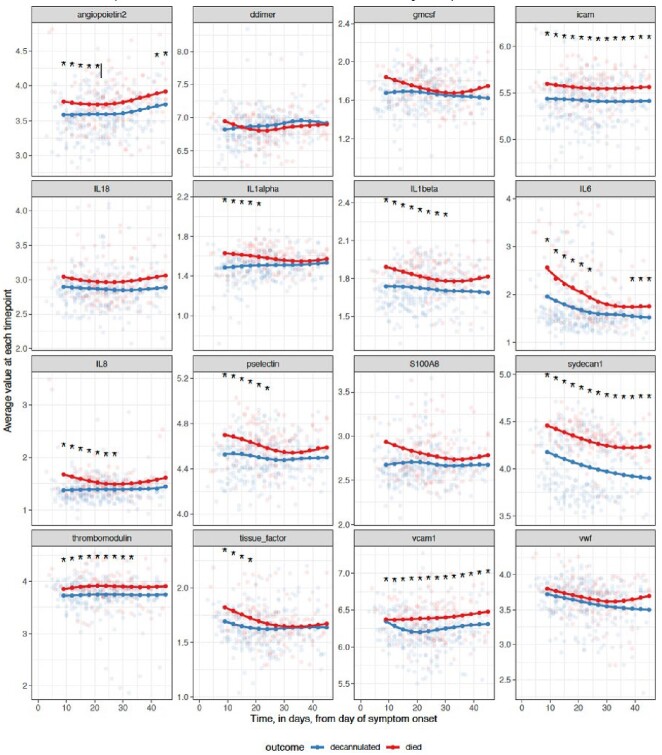
Non-parametric mixed model of 16 thromboinflammatory markers over time, separated by patient outcome. Asterixis represent significant differences (p<0.05) between patients who lived vs died. Blue: patients who were decannulated. Red: patients who died.

**Figure 2:**
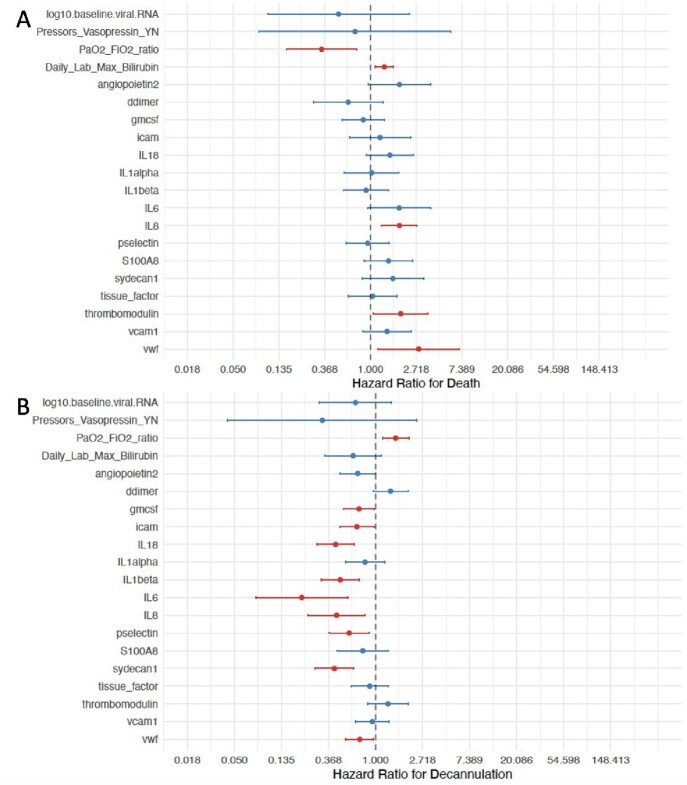
Cox regression models controlling for sex, smoking status, and time from symptom onset to cannulation. A) Hazard ratio of clinical, laboratory and thromboinflammatory measures for death. B) Hazard ratio of clinical, laboratory and thromboinflammatory measures for decannulation. Red bars indicate variables where the 95% confidence interval did not cross 1.

**Figure 3:**
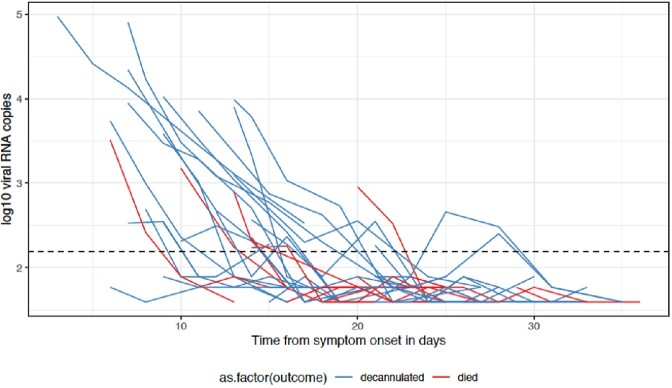
SARS-CoV-2 RNA viral copy number in plasma decreases over time. Lines represent individual patients. Dashed line represents limit of detection. Blue: patients who were decannulated. Red: patients who died.

**Conclusion:**

We found there was evidence for increased thromboinflammation in patients with COVID-19 on ECMO who died as compared to those who were decannulated. Multiple demographic and laboratory risk factors were also identified. These findings are important for understanding the pathogenesis of severe COVID-19 as well prognostication in the most critically ill patients and allocation of the scarce resource of ECMO support.

Funded by NCI Contract No. HHSN261201500003I

**Disclosures:**

**All Authors**: No reported disclosures

